# *Brucella* Strains at the Human–Animal Interface in Benin, Nigeria, Niger, Burkina Faso, and Togo: A Scoping Review

**DOI:** 10.1155/vmi/4521140

**Published:** 2026-11-01

**Authors:** Arnaud Soha, Beau-Gard Hougbenou, Gildas Hounmanou, Lauriano Houngbo, Horace Gankoutin, Gracia Toton, Brice Boris Legba, Victorien Dougnon

**Affiliations:** ^1^ Research Unit in Applied Microbiology and Pharmacology of Natural Substances Polytechnic School of Abomey-Calavi University of Abomey-Calavi Godomey Benin uac.bj

**Keywords:** *Brucella*, brucellosis, molecular epidemiology, One Health, West Africa

## Abstract

**Background:**

Brucellosis is a widespread zoonotic disease with significant impacts on both animal health and public health. This scoping review aimed to synthesize the available data on circulating *Brucella* species and laboratory identification methods in brucellosis research in Benin, Burkina Faso, Niger, Nigeria, and Togo.

**Methods:**

Article selection and examination followed the PRISMA extension for scoping reviews (PRISMA‐ScR) guidelines. Eligible studies published between 2010 and 2025 were identified through systematic searches of PubMed and Google Scholar using predefined eligibility criteria. A total of 162 articles were identified; after removing 25 duplicate records and 57 records published before 2010, 80 records were screened by title and abstract. Of these, 28 were excluded, leaving 52 reports assessed for eligibility. After full‐text reading, 8 reports were excluded, leaving 44 studies included in this review. Data extracted from each study included country, host species, sample type, identified *Brucella* species or biovars (when available), laboratory diagnostic methods, and study characteristics. Data were analyzed and visualized using R Version 4.5.2 with the tidyverse and ggplot2 packages.

**Results:**

We included 44 studies, primarily from Nigeria (91%; 40/44). Studies from Nigeria, Burkina Faso, Togo, and Niger met inclusion criteria; no eligible studies were found from Benin. Genus‐level identification (*Brucella* spp.) was reported in 75% (33/44) of studies, while species‐level identification was limited to 25% (11/44). *Brucella abortus* was the most commonly identified species (18%; 8/44). Serological methods predominated, used exclusively in 95.5% of studies (42/44), whereas molecular methods (PCR or qPCR) were used in only 2 studies (4.5%). Most studies (79.5%; 35/44) focused exclusively on animal samples, with limited integration of human (9.1%; 4/44) or food (2.3%; 1/44) samples; a further 4 studies (9.1%) combined animal samples with either human or food samples.

**Conclusions:**

Current evidence indicates that brucellosis surveillance remains largely dependent on serological diagnosis and animal‐based investigations, with limited application of molecular strain characterization and integrated human–animal surveillance. Strengthening laboratory capacity for molecular diagnostics, expanding genomic characterization of circulating *Brucella* species, and implementing harmonized One Health surveillance systems are essential to improve epidemiological understanding and guide effective brucellosis control in the region.

## 1. Introduction

Brucellosis is a globally significant endemic bacterial zoonotic disease and remains a major public health and veterinary concern, particularly in low‐ and middle‐income countries [1]. The epidemiology of human brucellosis, the most common zoonotic infection worldwide, has changed markedly over the past decade owing to various sanitary, socioeconomic, and political factors together with the evolution of international travel, underscoring the need for continued awareness of its evolving global distribution to guide appropriate interventions by international public‐health organizations [2]. Caused by bacteria of the genus *Brucella* [3], the disease affects a wide range of domestic and wild animals and can be transmitted to humans through direct contact with infected animals or indirectly via the consumption of contaminated animal products such as unpasteurized milk and dairy products [4]. At the human–animal interface, brucellosis represents a neglected zoonotic disease with significant socioeconomic and health impacts [5, 6]. As with other neglected zoonotic diseases, brucellosis persists largely outside formal surveillance systems, which leads to a substantial underestimation of its true incidence and burden and, in turn, to continued neglect by policymakers and funding agencies [7].

In West Africa, the rapidly increasing demand for dairy products is occurring in a context marked by a critical lack of up‐to‐date and reliable data on brucellosis burden, coupled with limited access to affordable diagnostic tools and effective vaccines [6]. High‐quality epidemiological research is essential for an accurate assessment of disease burden in regions such as Africa where data remain scarce, and an integrated One Health approach to surveillance, combining human and veterinary health services, would improve understanding of disease dynamics at the human–animal interface while enabling a more cost‐effective use of available resources [8]. Countries such as Benin, Togo, Nigeria, Niger, and Burkina Faso are characterized by extensive livestock production systems, close human–animal interactions, limited veterinary surveillance, and widespread informal trade of animal products [9]. These factors create favorable conditions for *Brucella* spp. maintenance and transmission both among animal populations and from animals to humans [10]. Human brucellosis is often underdiagnosed due to its nonspecific clinical presentation, which includes prolonged fever, fatigue, arthralgia, and musculoskeletal complications, and because of limited access to reliable diagnostic tools [9].

Livestock production in Benin, Nigeria, Niger, Burkina Faso, and Togo is dominated by interconnected pastoral, agropastoral, and mixed crop–livestock systems, with substantial livestock mobility across borders. Zoma‐Traoré et al. [11] documented diverse cattle production systems in Burkina Faso, while Ducrotoy et al. [12] highlighted the importance of livestock production and brucellosis in Nigeria. Across West and Central Africa, Musallam et al. [13] documented brucellosis risks within dairy production and milk supply chains. The selection of these countries is justified by their geographical proximity, shared livestock production, and transboundary animal movements, which facilitate pathogen circulation and support a regional One Health perspective.

Several *Brucella* species and biovars have been reported worldwide, with *Brucella abortus*, *Brucella melitensis,* and *Brucella suis* being the species most frequently associated with human infections [14]. The distribution of *Brucella* species and strains varies geographically and is influenced by host species, livestock production systems, husbandry practices, and surveillance capacity [15]. In West Africa, available studies indicate heterogeneous circulation of *Brucella* among cattle, small ruminants, horses, pigs, and humans; however, the evidence remains fragmented and largely derived from studies using diverse diagnostic and strain‐typing approaches [16].

Traditional phenotypic and serological methods have long been used for the detection and identification of *Brucella* spp., yet these approaches have important limitations in terms of sensitivity, specificity, and strain discrimination [17, 18]. Although molecular techniques such as PCR‐based assays, multilocus sequence typing (MLST), and whole‐genome sequencing (WGS) provide higher resolution for strain characterization, their application remains limited in many West African settings [16]. Consequently, comprehensive information on circulating *Brucella* strains at the human–animal interface in the region is lacking.

Given these gaps, a systematic mapping of existing evidence is needed. This scoping study aims to collate and synthesize available research on *Brucella* strains reported at the human–animal interface in Benin, Togo, Nigeria, Niger, and Burkina Faso. Specifically, it seeks to describe the diversity of *Brucella* species and strains identified, the populations and sample types studied, and the diagnostic and characterization methods employed [19]. By providing an overview of the current state of knowledge, this scoping review aims to synthesize the available data on circulating *Brucella* species and laboratory identification methods in brucellosis research in Benin, Burkina Faso, Niger, Nigeria, and Togo.

## 2. Methods

We conducted a preliminary search in PubMed and Google Scholar on September 19, 2025, to identify studies published between January 2010 and September 2025 that identified *Brucella* spp. in Benin and neighboring countries (Burkina Faso, Niger, Nigeria, and Togo). The search strategy combined the following keywords: “Brucella,” “human AND Brucella,” “animal AND Brucella,” and “human AND Brucella AND West Africa.” These keywords were used to formulate search queries for the scientific databases. Only articles published in English and French were considered. Eligible studies included original research reporting the detection or identification of *Brucella* spp. from human, animal, or environmental samples using serological, bacteriological, molecular, or other laboratory diagnostic methods. Reviews, conference abstracts, editorials, duplicate publications, studies conducted outside the target countries, studies published before 2010, and articles lacking sufficient methodological information or relevant results were excluded. The study selection process followed the PRISMA extension for scoping reviews (PRISMA‐ScR) recommendations [20]. A total of 162 records were identified through database searches (Figure 1). After removing duplicates (25), 137 records remained. Of these, 57 publications were excluded because they were published before 2010, leaving 80 records for title and abstract screening. Out of these, 52 full‐text articles were assessed for eligibility, and 8 studies were excluded for predefined reasons. Ultimately, 44 studies met the eligibility criteria and were included in the analysis. For each included study, data were extracted into Microsoft Excel (Supporting File 1, Database.xlsx), including the country, host species, sample type, *Brucella* species or biovar (when available), laboratory identification method, and other relevant study characteristics. Two independent reviewers screened titles, abstracts, and full texts; disagreements were resolved by consensus with a third reviewer, and the relevant variables were coded based on the research questions. The data were summarized using descriptive statistics and visualized in R Version 4.5.2 using the tidyverse and ggplot2 packages. As this was a scoping review, no meta‐analysis was performed.

**FIGURE 1:**
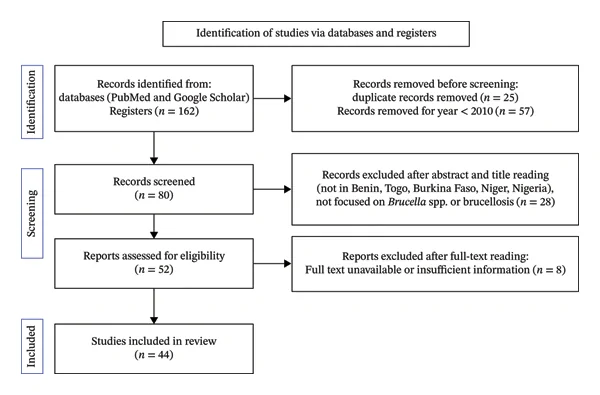
Article selection flowchart according to PRISMA extension for scoping reviews (PRISMA‐ScR).

## 3. Results

Figure 2 shows the distribution of included studies according to their year of publication between 2010 and 2025. This temporal distribution allows us to assess the evolution of scientific interest in the characterization of *Brucella* strains in the countries studied. The year 2016 had the most publications (7 studies), followed by 2012, 2013, and 2017, each with 5 studies. An emerging but still developing scientific dynamic is evident. This suggests that molecular surveillance of *Brucella* in these countries remains in a developmental phase and requires sustained support.

**FIGURE 2:**
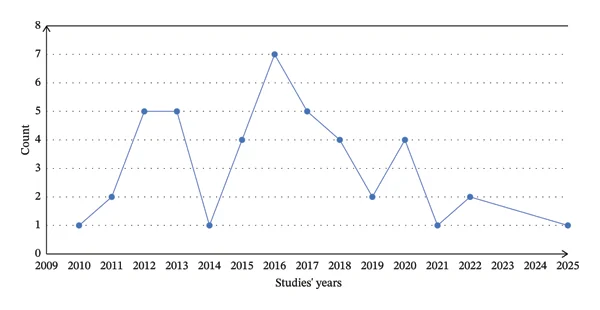
Included study year distribution.

The quantitative distribution of publications included in the review according to their geographic origin shows that four countries appear in this distribution: Nigeria, Burkina Faso, Togo, and Niger. Nigeria accounted for approximately 91% (40/44) of all included studies. This proportion indicates that most of the data available in the literature on *Brucella* strains in the study area originate from Nigeria. The observed distribution highlights a high concentration of publications in a single country, with very limited representation from Burkina Faso (2 studies), Togo and Niger (1 study each). No studies from Benin were identified for the period considered.

Figure 3A highlights a marked predominance of identification at the genus level. Approximately 75% (33/44) of the included studies report identification only as *Brucella* spp., without further specification at the species level. In contrast, about 25% (11/44) of the studies performed identification at the species level. This distribution indicates that, in the majority of the analyzed works, the characterization of isolates or positive samples was limited to confirming membership in the genus *Brucella*. Specific identification allowing differentiation between the various species within the genus remains in the minority. No significant proportion of studies appear to have reported identification at an infraspecific level (biovar, genotype, or sequence type), meaning that taxonomic resolution remains essentially limited to the first two levels: genus and species. Figure 3B details the *Brucella* taxa mentioned in the included studies. The majority of cases correspond to generic identifications under the name *Brucella* spp., representing approximately 75% (33/44) of the reported data. Among the studies that performed a specific identification, *Brucella abortus* appears as the most frequently identified species, representing approximately 18% (8/44) of documented cases. This species is the main one specifically mentioned in the results. *Brucella melitensis* is reported in a smaller proportion, at around 5% (2/44) of cases. *Brucella canis, Brucella abortus* biovar 3a, and *Brucella abortus* biovar 1 are each reported only marginally, at around 2% (1/44) of cases each. The observed distribution thus shows a significant concentration of data around two main categories: unspecified identifications (*Brucella* spp.) and specific identifications dominated by *Brucella abortus*.

**FIGURE 3:**
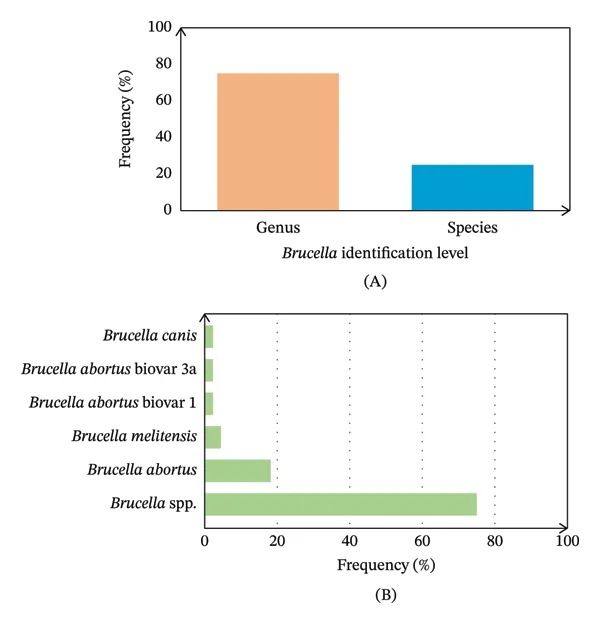
Distribution of level of strain identification in studies. (A) Level of strain identification. (B) Distribution of species identified in the included studies.

Studies used various sample types for brucellosis detection. Three groups were identified: animal, human, and food. The animal group was the largest and most diverse, comprising mainly livestock (cattle, sheep, goats, and camelids), draught animals (donkeys and horses), and domestic animals such as dogs. Samples were taken from live animals and livestock products. These consisted primarily of blood samples, animal carcasses, placentas, and aborted fetuses. Human samples were collected generally, but also from specific groups such as slaughterhouse workers and febrile patients. Food samples consisted mainly of milk. The majority of samples came from animals (35 out of 44 studies), indicating that most studies focused on animal reservoirs. Human samples are significantly underrepresented, at 4 out of 44, while food samples are very rare (1/44). This distribution reveals a strong imbalance in sampling sources, with animal research being predominant. There is also a predominance of studies using only animal samples. Studies based exclusively on human samples are few, and very few (4 out of 44) adopt an integrated approach including animal samples combined with human or food samples. These results indicate that the literature is heavily oriented towards animal reservoirs, while integrated approaches involving multiple sources (human and animal) remain limited. This situation suggests a lack of studies adopting a “One Health” perspective, which could limit the overall understanding of the transmission dynamics between animals, humans, and the food chain.

Figure 4 illustrates the distribution of strain identification methods in the analyzed studies. A clear predominance of serological methods is observed. This indicates that the majority of studies still favor serological approaches for strain identification, likely due to their accessibility, relatively low cost, and historical use in diagnostic laboratories. The vast majority of studies exclusively use serological methods. Studies using only molecular methods are very rare, and those combining molecular and serological methods are also very few. The molecular methods were based on PCR and qPCR. For those studies, a *Brucella* genus primer pair (forward: 5′‐CATATCGTTGCGCGTAAGGA‐3′ and reverse: 5′‐GAAACGCGCTTGCCTTTC‐3′) was used. Overall, these results reveal a strong reliance on serological approaches in the analyzed literature, with the adoption of molecular tools remaining very limited. This situation may limit taxonomic resolution and the ability to accurately characterize strains, highlighting the need to integrate more modern molecular methods to improve identification accuracy and the understanding of pathogen diversity.

**FIGURE 4:**
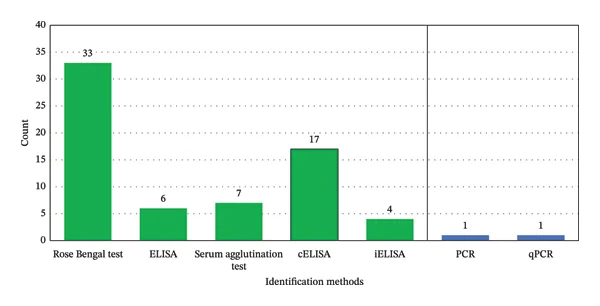
Distribution of strain identification methods. Studies could report more than one diagnostic method; therefore, percentages/frequencies are not mutually exclusive. cELISA, competitive ELISA; iELISA, indirect ELISA.

## 4. Discussion

The present analysis highlights a fundamental limitation in the diagnostic paradigm of brucellosis in West Africa, characterized by a predominant focus on detection rather than in‐depth taxonomic characterization. While genus‐level identification (reported as *Brucella* spp.) is sufficient to confirm infection, it remains insufficient to decipher transmission dynamics, host specificity, and zoonotic risk patterns [21]. This limitation may also contribute to biased epidemiological interpretations, including a potential overestimation of *Brucella abortus* due to methodological constraints. The predominance of genus‐level identification reflects persistent diagnostic and methodological challenges, particularly in low‐ and middle‐income settings. As a result, the lack of strain‐level data obscures the true diversity of circulating *Brucella* and restricts the development of targeted control strategies.

This imprecision largely results from the widespread use of serological methods, which remain the cornerstone of brucellosis diagnosis in the region due to their accessibility and low cost [21–23]. This trend is widely reported and largely attributed to the use of serological assays such as Rose Bengal test and enzyme‐linked immunosorbent assay (ELISA), as well as conventional PCR methods that confirm genus presence but lack discriminatory power at the species or strain level [12, 23]. Unlike molecular approaches, serology does not allow for phylogenetic analysis or transmission network reconstruction [23, 24]. Although these tools are useful for surveillance, they limit epidemiological interpretation, including the assessment of host specificity, transmission pathways, and zoonotic risks. The relatively low proportion of species‐level identification, dominated by *Brucella abortus*, is consistent with its established role as the primary agent of bovine brucellosis in endemic regions [25]. However, this apparent dominance may also reflect diagnostic bias rather than true epidemiological distribution.

In this context, the near‐absence of *Brucella melitensis* in the reviewed literature warrants particular attention. Within our dataset, *B. melitensis* was reported in only two studies, both conducted in Nigeria, including a geospatial investigation in Katsina and Sokoto States that specifically targeted small ruminant populations [26]. This scarcity is unlikely to reflect the true epidemiological situation, given the well‐established importance of *B. melitensis* as the principal cause of caprine and ovine brucellosis worldwide and its recognized status as the most pathogenic *Brucella* species for humans [27]. Rather, it more plausibly reflects a long‐standing research and surveillance bias toward cattle, which has left small ruminant brucellosis comparatively neglected across Sub‐Saharan Africa despite the central role of sheep and goats in regional livestock economies and household food security [27]. This pattern is consistent with the predominance of cattle‐focused sampling identified elsewhere in the present review, which likely compounds the underdetection of *B. melitensis* by directing surveillance effort disproportionately toward bovine hosts.

Beyond species identification, the near‐absence of infraspecific characterization represents a critical gap. This limitation is particularly concerning given the emergence of atypical *Brucella* strains and increasing reports of cross‐species transmission events [16, 28]. Although traditional bacteriological methods, Bruce‐ladder PCR, and other bio‐ and genotyping approaches can be used to identify *Brucella* strains, their application remains limited in the region. More advanced tools such as multilocus variable number tandem repeat analysis (MLVA), single‐nucleotide polymorphism (SNP)‐based typing, and WGS are increasingly recommended for high‐resolution epidemiological tracing, outbreak investigation, and One Health surveillance [29–32]. Their underutilization constitutes a major bottleneck. In particular, the absence of WGS isolates the region from current international genomic surveillance standards, leading to an underestimation of pathogen diversity and limiting the development of effective, targeted control strategies.

The distribution of sampling sources further reveals conceptual limitations in current research approaches. The predominance of animal studies, combined with the underrepresentation of human and food samples, reflects a siloed research paradigm [33]. Although the focus on animal reservoirs is justified by the zoonotic nature of brucellosis [23, 34], the lack of cross‐sectoral integration limits the ability to reconstruct transmission chains. Notably, very few studies adopt a truly integrated One Health approach that simultaneously considers animal, human, and environmental compartments. This gap is particularly striking given its importance for zoonotic disease surveillance [35]. Without such integration, critical interfaces such as foodborne transmission and occupational exposure remain poorly characterized [36, 37]. Consequently, current evidence is insufficient to support comprehensive risk assessments or inform effective cross‐sectoral interventions.

Taken together, these findings suggest that the observed limitations are not isolated issues but rather reflect broader structural gaps in regional surveillance systems [33]. The region is characterized by limited laboratory infrastructure, restricted access to advanced technologies, and a lack of coordinated research networks. These constraints affect the quantity of available data and its quality, resolution, and representativeness. Furthermore, the absence of standardized protocols and harmonized data systems reduces comparability across studies and countries, thereby weakening the overall evidence base. Addressing these challenges will require a transition from fragmented, project‐based research toward integrated and systemic surveillance approaches.

From a strategic perspective, strengthening diagnostic capacity through the integration of molecular and genomic tools should be considered a priority. Even the gradual adoption of PCR‐based diagnostics could significantly improve species‐level identification [38], while the targeted implementation of WGS in regional reference laboratories would enable precise strain tracking. Importantly, these efforts must be embedded within a comprehensive One Health framework, ensuring coordinated collection and analysis of human, animal, and environmental data. High‐quality, formally trained epidemiological research, coupled with closer collaboration between human and veterinary health services, would strengthen data reliability while making more efficient use of limited resources [8]. Regional collaboration will be essential to address transboundary transmission dynamics and to harmonize surveillance strategies across countries [33]. The development of regional surveillance platforms combining standardized protocols, shared data systems, and coordinated sampling strategies could significantly enhance the robustness and reliability of epidemiological data.

### 4.1. Limitations of the Review

This scoping review has several limitations that should be considered when interpreting its findings. First, the evidence identified in this review is subject to potential publication bias. Studies reporting positive findings or higher prevalence estimates may be more likely to be published than studies reporting negative or inconclusive results. Consequently, the mapped literature may not fully represent the underlying epidemiology of brucellosis in the countries considered. This is particularly relevant for settings where surveillance and research activities are limited.

Second, language bias cannot be excluded. Although the search strategy was designed to identify relevant literature from the countries of interest, the review may have underrepresented studies published in languages other than those covered by the search strategy. This limitation is particularly relevant in a multilingual region where potentially relevant evidence may be reported in languages other than English or French, or in local or regional sources. Future reviews could improve the comprehensiveness of the evidence map by searching additional regional databases and gray literature.

Third, the number and distribution of available studies varied substantially between countries. The absence or limited number of studies from a country should therefore not be interpreted as evidence of the absence of brucellosis. In particular, the lack of identified studies from Benin and the single study from Niger may reflect under‐investigation, limited publication activity, or gaps in surveillance rather than a true absence of disease. Similarly, the predominance of studies from Nigeria may reflect its larger research output and population size rather than a proportionally higher burden of brucellosis.

Fourth, substantial heterogeneity was observed in study populations, sample types, diagnostic methods, and reporting practices. The frequent use of serological methods and the limited application of molecular techniques restrict direct comparison of findings across studies and may limit species‐level characterization. In addition, several studies used multiple diagnostic methods, further complicating direct comparison of diagnostic frequencies. Because this was a scoping review, no quantitative synthesis or pooled prevalence estimate was attempted, and the findings should therefore be interpreted as a map of the available evidence rather than as an estimate of regional disease prevalence.

Finally, the conclusions of this review are constrained by the information reported in the primary studies. Important dimensions of brucellosis epidemiology and control, including antimicrobial resistance, vaccination coverage and effectiveness, national surveillance systems, occupational exposure, and genomic characterization, were poorly documented or not investigated in the included literature. These gaps represent limitations of the available evidence base rather than variables that could be systematically assessed by this review. Consequently, further integrated One Health studies combining standardized diagnostic approaches, epidemiological surveillance, and molecular characterization are needed to improve the understanding of brucellosis in Benin and neighboring countries.

## 5. Conclusion

In conclusion, this scoping review shows that evidence on *Brucella* identification in Benin and neighboring West African countries remains limited and unevenly distributed, with a strong reliance on serological methods and limited use of molecular approaches. Beyond descriptive findings, the results highlight a deeper issue: the absence of a coherent, high‐resolution, and integrated surveillance framework capable of capturing the complex epidemiology of brucellosis at the human–animal–environment interface. Overall, brucellosis research in the region remains largely descriptive, emphasizing the urgent need to strengthen laboratory capacity and integrate advanced molecular and genomic tools. In the short term, laboratory capacity should be strengthened through standardized diagnostic protocols and greater access to PCR‐based identification; in the medium term, molecular characterization using approaches such as MLVA, MLST and, where feasible, WGS should be progressively integrated into surveillance. Regional collaboration among laboratories and veterinary and public health institutions should support harmonized surveillance and cross‐border data sharing, particularly given the limited evidence identified from Benin and Niger. Research priorities should include species and strain‐level characterization, standardized surveillance across human and animal populations, and investigation of transmission pathways at the human–animal interface. Such improvements are essential to enhance epidemiological understanding, guide control strategies, and support evidence‐based policy decision‐making.

## Author Contributions

Arnaud Soha conceptualized the idea. Beau‐Gard Hougbenou collected data. Lauriano Houngbo, Gracia Toton, and Horace Gankoutin drafted the manuscript. Arnaud Soha, Gildas Hounmanou, Brice Boris Legba, and Victorien Dougnon took part in editing, preparing, and critically checking this manuscript.

## Funding

No funding was received for this study.

## Disclosure

The lead author affirms that this manuscript is an honest, accurate, and transparent account of the study being reported; that no important aspects of the study have been omitted; and that any discrepancies from the study as planned have been explained.

## Conflicts of Interest

The authors declare no conflicts of interest.

## Supporting Information

Additional supporting information can be found online in the Supporting Information section.

## Supporting information


**Supporting Information** Supporting File 1 (Database.xlsx): A comprehensive spreadsheet containing a curated dataset of 44 peer‐reviewed epidemiological studies on brucellosis across Nigeria, Burkina Faso, Togo, and Niger. It includes structural data on article titles, country of origin, publication years, matrices/hosts sampled, targeted bacterial species, and the specific diagnostic or serological methods employed.

## Data Availability

The data supporting the findings of this study are available within the article and its supporting information file (Supporting File 1, Database.xlsx).
